# Overexpression of microRNA-9 enhances cisplatin sensitivity in hepatocellular carcinoma by regulating EIF5A2-mediated epithelial-mesenchymal transition

**DOI:** 10.7150/ijbs.32460

**Published:** 2020-01-16

**Authors:** Ying Bao, Yibo Zhang, Yongliang Lu, Huihui Guo, Zhaohuo Dong, Qiuqiang Chen, Xilin Zhang, Weiyun Shen, Wei Chen, Xiang Wang

**Affiliations:** 1 Key Laboratory for Translational Medicine, First Affiliated Hospital, Huzhou University, the First People's Hospital of Huzhou, huzhou 313000,China; 2 Department of Hepatobiliary and Pancreatic Surgery, Second Affiliated Hospital of Zhejiang University School of Medicine, Hangzhou, 310009, China; 3 Department of medicine,Huzhou University, huzhou 313000,China; 4 Cancer Institute of Integrated traditional Chinese and Western Medicine, Key laboratory of cancer prevention and therapy combining traditional Chinese and Western Medicine, Zhejiang Academy of Traditional Chinese Medicine, Hangzhou, Zhejiang, 310012, China; 5 Department of Medical Oncology, Tongde hospital of Zhejiang Province, Hangzhou, Zhejiang, 310012, China

**Keywords:** miR-9, EIF5A2, chemoresistance, epithelial-mesenchymal transition

## Abstract

We investigated the role of microRNA (miR)-9 in modulating chemoresistance in hepatocellular carcinoma (HCC) cells. MiR-9 was overexpressed or knocked down in HCC cell lines. Cell viability, cell proliferation, the expression of EIF5A2 and the epithelial-mesenchymal transition (EMT)-related proteins were examined. HCC cells overexpressing miR-9 were more sensitive to cisplatin; miR-9 knockdown yielded the opposite result. The *in vivo* nude mouse HCC xenograft tumors yielded the same results. EIF5A2 was identified as a potential target of miR-9, where miR-9 regulated EIF5A2 expression at mRNA and protein level. EIF5A2 knockdown reversed miR-9 inhibition-mediated cisplatin resistance. Altering miR-9 and EIF5A2 expression changed E-cadherin and vimentin expression. Furthermore, EIF5A2 mediated miR-9 EMT pathway regulation, indicating that miR-9 can enhance cisplatin sensitivity by targeting EIF5A2 and inhibiting the EMT pathway. Targeting miR-9 may be useful for overcoming drug resistance in HCC.

## Introduction

Hepatocellular carcinoma (HCC) is one of the most common cancers and is currently the third leading cause of cancer death worldwide. HCC incidence is highest in Asia, and more than 50% of cases are in China 1, 2. Systematic chemotherapy, especially molecular targeted agents such as sorafenib, plays a key role in treating HCC, especially for patients with advanced disease; however, the median survival time of patients was extended by only three months 3, 4. Cisplatin is extensively applied as the first-line chemotherapeutic agent for treatment in the clinic against a wide spectrum of cancers, including liver cancer, ovarian and small cell lung cancer^5^, ^6^. However, due to severe side effects and drug resistance, the clinical applications of DDP are largely limited 7. Chemoresistance limits the use of chemotherapy in HCC. To date, the underlying mechanisms causing chemoresistance in HCC are largely unknown. Accordingly, uncovering the cellular and molecular mechanisms underlying the development of HCC chemoresistance is essential for developing effective chemotherapeutics and improving the prognosis of patients with HCC.

MicroRNAs (miRNAs) are small, noncoding, ~22-nucleotide RNAs that regulate gene expression by interacting with the 3′ untranslated regions (3′ UTRs) of the target mRNA to induce mRNA degradation or suppress mRNA translation 8, 9. Accumulating evidence indicates that miRNAs play critical roles in cell growth, differentiation, apoptosis, metastasis, and drug resistance, and are involved in many diseases, including cancer 10. Several studies have shown a correlation between miRNAs and HCC chemoresistance. MiR-7 regulates sorafenib sensitivity by targeting the GAS6/TYRO3 (growth arrest-specific 6/TYRO3 protein tyrosine kinase) axis 11. Jin et al. found that miR-26a/b can sensitize HCC to chemotherapy by suppressing the expression of the autophagy initiator ULK1 (unc-51-like autophagy-activating kinase 1) 12. Moreover, inhibiting miR-205-5p reversed HCC resistance to 5-fluorouracil (5-FU) by regulating the PTEN/JNK/ANXA3 (phosphatase and tensin homolog/Janus kinase/annexin A3) pathway 13.

The miRNA expression patterns and functions in cancer can be tissue- and cell type-dependent. MiR-9 is involved in tumor proliferation and cancer cell autophagy, migration, and apoptosis, either as an oncogene or a tumor suppressor, based on the cancer type 14-18. There is no consensus on whether miR-9 functions as an oncogene or a tumor suppressor in HCC. Ding et al. found that over-expression of miR-9-5p promotes HCC cell migration and invasion by targeting E-cadherin 19. Sun et al. showed that upregulation of miR-9-5p enhances HCC cell migration and invasion through KLF17 (Krüppel-like factor 17) 20. In HCC samples, high miR-9 expression was an independent prognostic factor of decreased survival 21, 22. Higashi et al. showed that miR-9-3p plays a tumor suppressor role in HCC cells by targeting TAZ (tafazzin) 23. Zhang et al. found that miR-9 plays a tumor-suppressive role in HCC partially by regulating the IGF2BP1/AKT&ERK (insulin-like growth factor 2 mRNA-binding protein 1/AKT and extracellular signal-regulated kinase) axis 24. However, little is known about the role of miR-9 in HCC chemoresistance.

In the present study, we evaluated the biological effects of miR-9 on cisplatin sensitivity in HCC. We also explored the underlying mechanisms of miR-9 promotion of cisplatin sensitivity in HCC cells.

## Materials and methods

### Cell culture and chemical reagents

The human HCC cell lines Hep3B, HepG2, SNU387, and SNU449 were purchased from American Type Culture Collection (ATCC, Manassas, VA, USA) and cultivated according to ATCC's instructions. Huh7 cells were purchased from the Chinese Academy of Science Cell Bank (Shanghai, China). Hep3B, HepG2, and Huh7 cells were cultured in Eagle's minimum essential medium (EMEM, Gibco, Grand Island, NY, USA); the SNU387 and SNU449 cells were cultured in RPMI 1640 medium (Gibco, Grand Island, NY, USA). All mediums were supplemented with 10% fetal bovine serum (FBS, Gibco, Grand Island, NY, USA); all cells were maintained at 37°C in a humidified incubator with 5% CO_2_. Cisplatin was purchased from Selleck (Houston, TX, USA).

### Cell viability assay

To detect the relative cell viability, the cells were incubated in 10% Cell Counting Kit-8 (CCK-8) assay (Dojindo, Kumamoto, Japan) diluted in culture medium at 37°C for 2 h. The cell proliferation rates were determined at 48 h post-treatment of cisplatin, and the optical density at 450 nm (OD450) value was quantified on a microtiter plate reader (Dynex, Chantilly, VA, USA). All reactions were performed in triplicate.

### 5-Ethynyl-2′-deoxyuridine (EdU) staining

The cells were treated with miR-9 mimic, inhibitor, or negative control for 48 h and then with cisplatin (1 µg/ml) for 48 h. EdU staining was performed using a Click-iT EdU Imaging Kit (Invitrogen, Carlsbad, CA, USA) according to the manufacturer's instructions. The total number of cells (Hoechst 33342-positive cells) and the number of proliferating cells (EdU-positive cells) were recorded in three independent experiments, and the means were obtained. All reactions were performed in triplicate.

### Cell transfection

MiR-9 mimic, inhibitors, negative control, and eukaryotic translation initiation factor 5A2 (EIF5A2) small interfering RNA (siRNA) were synthesized by Shanghai GeneChem (Shanghai, China). HCC cells were seeded 1×10^5^cells/well into six-well plates, transfected with 100 nM miR-9 mimic, 100 nM inhibitor, 100 nM EIF5A2 siRNA, and 100 nM negative siRNA using Lipofectamine 2000 (Invitrogen) according to the manufacturer's instructions. The sequence of synthesis and EIF5A2 siRNA were as follows:

miR-9-5p mimic: Forward: 5'-UCUUUGGUUAUCUAGCUGUAUGA-3', Reverse: 5'-AUACAGCUAGAUAACCAAAGAUU-3'; miR-9-5p inhibitor: 5'-UCAUACAGCUAGAUAACCAAAGA-3'; EIF5A2 siRNA: Forward: 5'- GCAGACGAAAUUGAUUUCATT -3', Reverse: 5'- UGAAAUCAAUUUCGUCUGCTT -3'; Negative Control: Forward: 5'- UUCUCCGAACGUGUCACGUdTdT-3', Reverse: 5'-ACGUGACACGUUCGGAGAAdTdT-3'.

### Real-time PCR

Total RNA was extracted using TRIzol following the manufacturer's protocol (Invitrogen). Complementary DNA (cDNA) was synthesized using Moloney murine leukemia virus (M-MLV) reverse transcriptase (Invitrogen) from 2 μg total RNA. EIF5A2 expression was measured with an ABI7500 Fast system (Applied Biosystems, Foster City, CA, USA) and SYBR Green dye (TaKaRa Biotechnology, Dalian, China) according to the manufacturers' instructions. Glyceraldehyde-3-phosphate dehydrogenase (*GAPDH*) was used as the internal control, and the comparative threshold cycle (2^-∆∆Ct^) method was used for relative quantification. All reactions were performed in triplicate. The primer sequences were as follows:

miR-9-5p: 5'-TCTTTGGTTATCTAGCTGTATGA-3'; EIF5A2: Forward 5'-TATGCAGTGCTCGGCCTTG-3', Reverse 5'-TTGGAACATCCATGTTGTGAGTAGA-3'; U6: Forward 5'-CTCGCTTCGGCAGCACA-3', Reverse 5'-AACGCTTCACGAATTTGCGT-3'; β-Actin: Forward 5'- GACTTAGTTGCGTTACACCCTT-3', Reverse 5'-TTTTGACCTTGCCACTTCCA-3'.

### Western blotting

Protein was extracted from HCC cells with radioimmunoprecipitation assay (RIPA) lysis buffer (Beyotime, Shanghai, China) supplemented with a phenylmethylsulfonyl fluoride (PMSF) inhibitor (Beyotime), and quantitatively analyzed with a bicinchoninic acid (BCA) kit (Thermo Scientific, Waltham, MA, USA). The proteins were separated with 10% sodium dodecyl sulfate-polyacrylamide gel electrophoresis (SDS-PAGE), and then transferred to polyvinylidene fluoride (PVDF) membranes. The membranes were incubated with 5% non-fat milk containing 0.1% Tween 20 for 2 h at room temperature to block nonspecific binding. The membranes were incubated with primary antibodies against eIF5A2 (diluted 1:1000; Abcam(ab150439), Cambridge, MA, USA), β-actin(ab8226), E-cadherin(ab40772), vimentin (ab92547) (1:2000; Abcam) and GAPDH(Cell Signaling Technology, Inc.; cat no. 5174S; diluted 1:2,000)at 4°C overnight. After washing with Tris-buffered saline containing Tween 20 (TBST), the membranes were incubated with the secondary antibody( diluted 1:5000; Abcam(ab97051)) for 1 h at 37°C. The proteins were visualized using an ECL Plus detection system (Millipore, Billerica, MA, USA) The bands analyzed by Western Blot were cropped from different parts of the same and the different gel. Respective images were cropped for figure implementation. All reactions were performed in triplicate.

### Nude mouse xenograft model

The experimental procedures were proved by the Medical Ethics Committee of the First Affiliated Hospital of Hu Zhou University and use of laboratory animals of the First Affiliated Hospital of Hu Zhou University, Huzhou, China, and conformed to the National Institutes of Health Guide for Care and Use of Laboratory Animals (NIH Publications, No. 8023, revised 1978). We declared that experimental protocols were approved by the Medical Ethics Committee of the First Affiliated Hospital of Hu Zhou University and we had provided the approval files. The experimental protocols are as follows: Female BALB/c nu/nu mice (4-5 weeks old) were used for the xenografting experiment. Huh7 cells (1 × 10^6^) were suspended in 100 µl phosphate-buffered saline (PBS) and injected subcutaneously in to the abdominal region of the mice. After 10 days, the tumors were 0.5 cm in diameter, and the tumors reached around 50-100 mm^3^, the mice were randomly divided in to four groups (n = 4 per group). In this experiment, we used miR-9 agomiR (RiboBio, Guangzhou, China, 5'-UCUUUGGUUAUCUAGCUGUAUGA-3'), a chemically modified miR-9 mimic with suitable pharmacokinetic properties for *in vivo* studies 25. The mice received multi-point intratumoral injections of miR-9 mimic (10 nmol), miR-9 mimic (10 nmol) plus cisplatin (5 mg/kg), cisplatin (5 mg/kg), or control (normal saline) on day 1, 3, 5, and 7 of the experiment. Tumor volumes were recorded every 2 days and calculated with the following formula. The tumor volume (mm3) was calculated according to the following formula: length × width^2^/2. The mice were sacrificed humanely on day 15 after treatment, and the resected tumors were weighed.

### Immunocytochemistry

Immunohistochemical staining was performed on paraffin-embedded mouse tissue sections (5 mm) to determine Ki-67 expression. The slides were incubated with anti-Ki-67 antibody (1:500, Abcam (ab15580)) overnight at 4°C. Horseradish peroxidase (HRP) Detection System (ZSGB-bio, Beijing, China) and diaminobenzidine (DAB) Substrate Kit (ZSGB- bio) were used as detection reagents. After counterstaining with hematoxylin (ZSGB-bio), the sections were dehydrated and mounted, and observed under light microscopy (Olympus, Tokyo, Japan). The positive rates were measured using Image-Pro Plus v. 6.0 software (Media Cybernetics, Bethesda, MD, USA). All reactions were performed in triplicate.

### Terminal deoxynucleotidyl transferase dUTP nick end labeling (TUNEL)

TUNEL was used to identify apoptosis in paraffin-embedded mouse tissue sections (5-mm) with an *in situ* cell death detection kit (Roche, Basel, Switzerland) according to the manufacturer's instructions. The apoptotic cells were observed under a light microscope (Olympus, Tokyo, Japan). The assay was independently repeated three times. The positive rates were measured using IPP 6.0 software. All reactions were performed in triplicate.

### Luciferase Reporter Assay

Wild type EIF5A2-3'UTR or mutant EIF5A2-3'UTR was structured by 3'-UTR of EIF5A2 that included the binding sequence for miR-9 or the mutated 3'-UTR. For the luciferase reporter assays, HEK293 cells were plated in 96-well plates and then transiently co-transfected with luciferase reporter vectors with miR-9a-mimic, miR-9 inhibitor or control using Lipofectamine 2000. After transfection for 48h, the luciferase reporter assay (Promega, USA) was used to measure the luciferase activity of the wild type or mutant EIF5A2 3'-UTR.

### Statistical analysis

The statistical significance of the results was determined using the Student *t*-test or one-way analysis of variance (ANOVA). P < 0.05 was considered statistically significant.

## Results

### HCC cell lines with high miR-9 expression had higher cisplatin sensitivity

**Figure 1:**
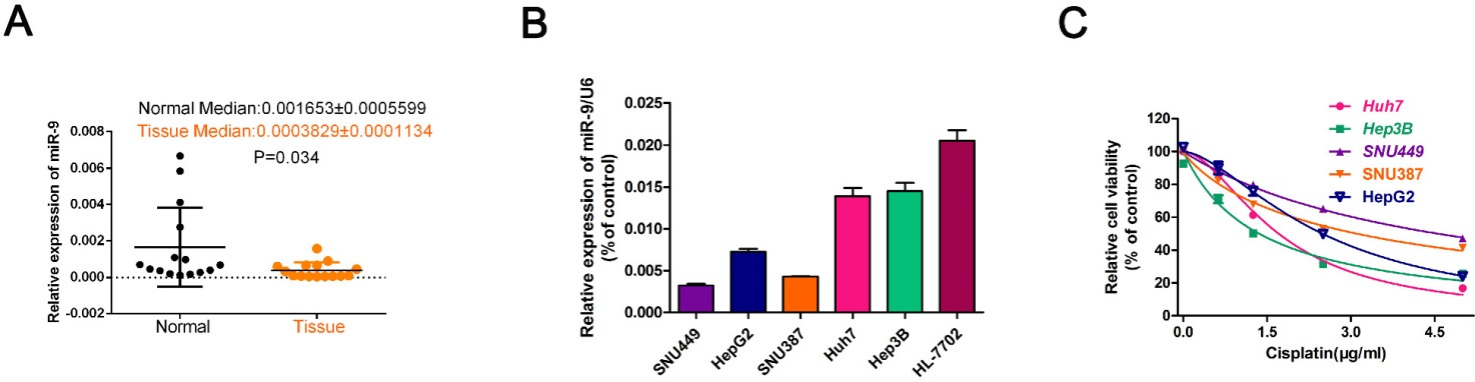
** miR-9 expression pattern in HCC cell lines and its relationship with cisplatin sensitivity.** (**A**) The miR-9 expression was detected in 20 paired HCC tissues and normal tissues. (**B**) Real-time PCR investigation of miR-9 expression in HCC cell lines and the normal hepatic cells HL-7702. (**C**) CCK-8 assay of HCC cell viability in the presence of cisplatin (0, 1.5, 3.0, 4.5 μg/ml).

To investigate the role of miR-9 in the cisplatin sensibility of HCC cell lines, firstly, we collected 20 paired HCC tissues and normal tissues and detected miR-9 expression. Compared with control, the level of miR-9 was decreased in tumor tissues in **Figure 1A**. Then, we tested miR-9 expression in five HCC cell lines and the normal hepatic cell HL-7702 by quantitative reverse transcription-PCR (qRT-PCR). The miR-9 expression levels in the HCC cell lines were as follows: Hep3B > Huh7 > HepG2 > SNU387 > SNU449 **(Figure 1B)**. Examination of the IC50 of cisplatin on HCC cells revealed an opposite trend compared to that of the miR-9 levels, i.e., SNU449 > SNU387 > HepG2 > Huh7 > Hep3B (IC50(μg/ml): SNU449,4.556; SNU387, 3.104; HepG2, 2.491; Huh7,1.706; Hep3B,1.356) **(Figure 1C, Table 1)**. Therefore, our results indicate that miR-9 expression levels may correlate positively with cisplatin sensitivity in HCC.

### miR-9 overexpression enhanced HCC cell cisplatin sensitivity *in vitro* and *in vivo*

**Figure 2:**
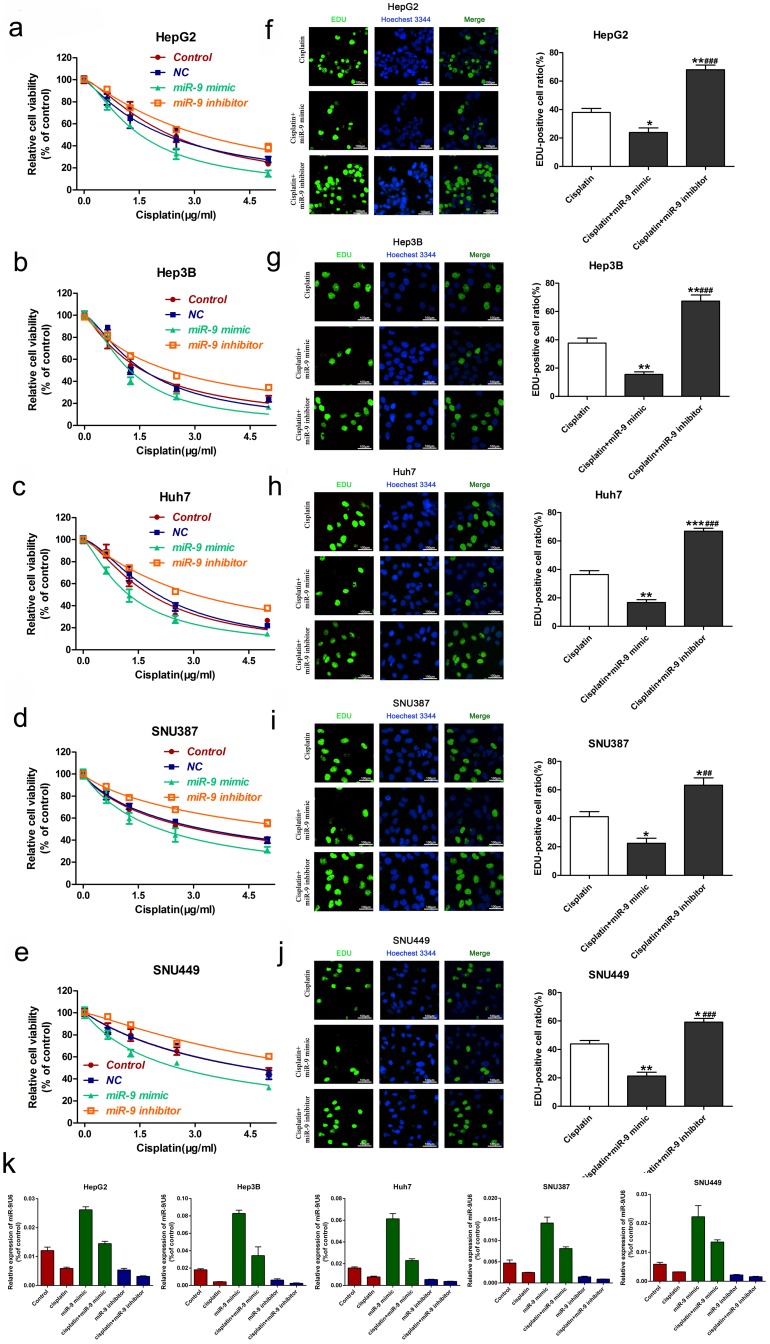
** Effect of miR-9 plus cisplatin on HCC cell proliferation and apoptosis combined *in vitro*.** (**A-E**) CCK-8 assay of the viability of HCC cells treated with miR-9 mimic, miR-9 inhibitor, or corresponding control RNAs in the presence of cisplatin (0, 1.5, 3.0, 4.5 μg/ml). (**F-J**) EdU assay of cell proliferation rate under cisplatin in HCC cell lines treated with miR-9 mimic, miR-9 inhibitor, or control RNAs. The EdU-positive cells were counted. *vs. cisplatin, ^#^vs. cisplatin plus miR-9 mimic, *P < 0.05, **P < 0.01, ***P < 0.001, ^##^P < 0.01, ^###^P < 0.001.(K).QRT-PCR of miR-9 level in HCC cell treated with miR-9 mimic , inhibitor or combined with cisplatin, *P<0.05.

To explore the role of miR-9 on cisplatin sensitivity in HCC, we used gain- and loss-of-function approaches *in vitro* and *in vivo*. The *in vitro* experiments showed that, in the presence of cisplatin, miR-9 knockdown HCC cell lines had the best viability compared to the other treatment groups; opposite results were observed in the miR-9 overexpression HCC cell lines **(Figure 2A-E, Table 2).** *In vitro*, EdU staining showed that after cisplatin exposure, HCC cells that had been transfected with miR-9 mimic had reduced cell proliferation rates compared with cells transfected with miR-9 inhibitor **(Figure 2F-J)**. The level of miR-9 after transfected with miR-9 mimic, inhibitor or plus with cisplatin for 48h was determined by qRT-PCR **(Figure 2K).**

**Figure 3:**
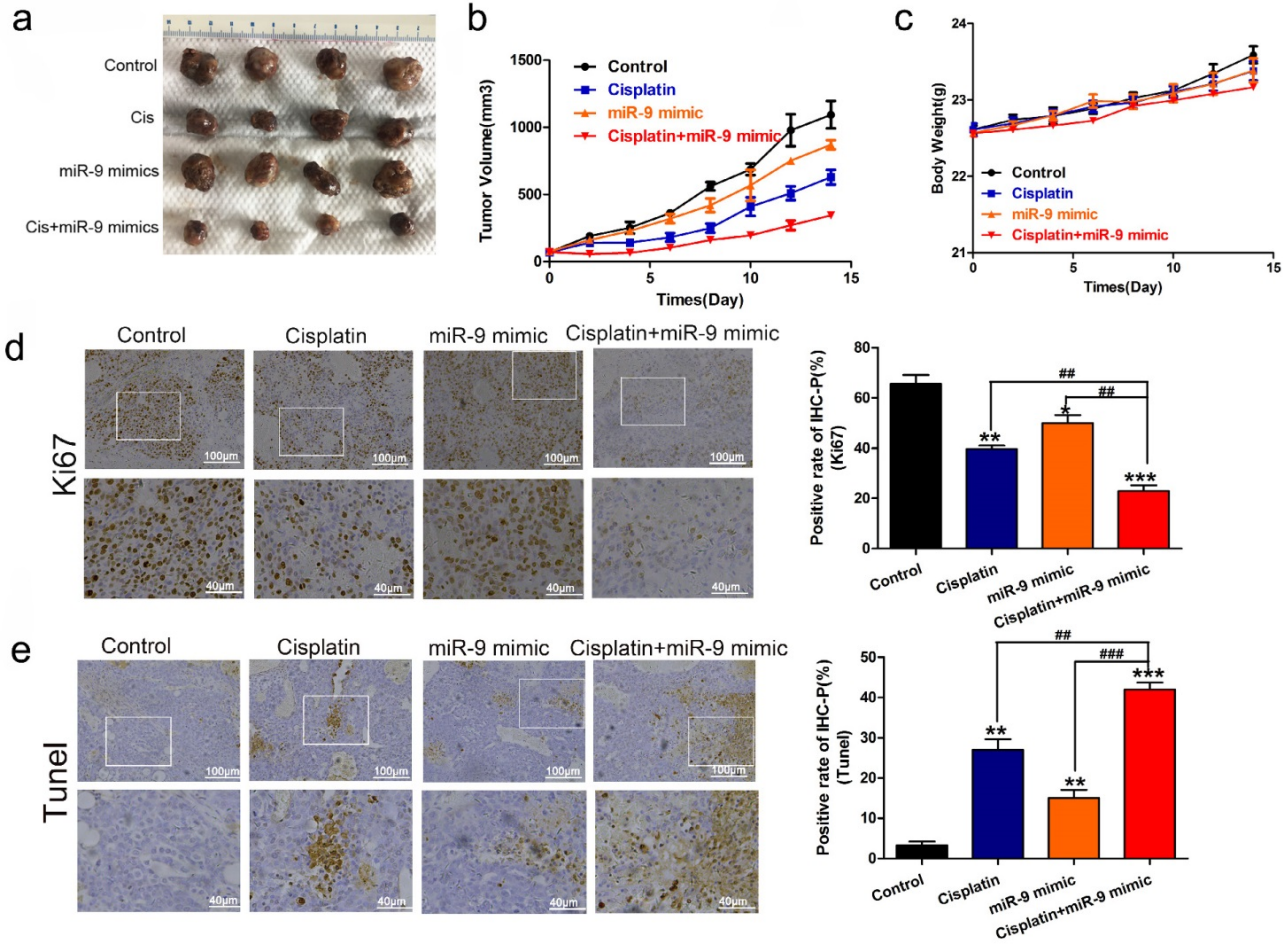
** Effect of miR-9 plus cisplatin on HCC cell viability and proliferation *in vivo*.** (**A**) Gross morphology of subcutaneous xenograft tumors. The tumors were treated with control, cisplatin, miR-9 mimic, or cisplatin plus miR-9 mimic. (**B**) Growth curves of xenograft tumors treated with control, cisplatin, miR-9 mimic, or cisplatin plus miR-9 mimic (n = 4 per group). *P < 0.05,***P < 0.001 vs Control. **(C)** The weights of mice were measured on day 0-15 which was treated with control, cisplatin, miR-9 mimic, or cisplatin plus miR-9 mimic. (**D**) Ki-67 staining in the treatment groups (×40 magnification) and rate of Ki-67 positive staining. Bottom panels depict the insets of the top panels (×100 magnification). (**E**) TUNEL of apoptosis in the treatment groups (×40 magnification). Bottom panels depict the insets of the top panels (×100 magnification). *vs. control, ^#^vs. cisplatin plus miR-9 mimic, *P < 0.05, **P < 0.01, ***P < 0.001, *^##^*P < 0.01, ^###^P < 0.001. IHC-P, immunohistochemistry-positive.

Next, we used nude mice tumorigenesis testing to explore the effect of miR-9 on cisplatin sensitivity *in vivo*. The mice were treated with vehicle alone, cisplatin alone, miR-9 mimic alone, or cisplatin plus miR-9 mimic. Tumor growth was statistically significantly delayed in the cisplatin plus miR-9 mimic group **(Figure 3A)**, with reduced tumor volume compared with vehicle, cisplatin alone, or miR-9 mimic alone groups** (Figure 3B)**. Moreover, the body weights on day 15 after tumor cell injection in the cisplatin plus miR-9 mimic group were statistically significantly lower than that of the other three groups** (Figure 3C),** and the tumor weight was also reduced in cisplatin plus miR-9 mimic groups **(Figure S1).** Ki-67 staining showed significantly decreased tumor cell proliferation rates in the cisplatin plus miR-9 mimic group compared to the other three groups **(Figure 3D)**; TUNEL examination showed higher tumor cell apoptosis in the cisplatin plus miR-9 mimic group **(Figure 3E)**. These data support the idea that miR-9 is a potent cisplatin sensitizer *in vitro* and *in vivo*.

### miR-9 targeted EIF5A2 in HCC cells

**Figure 4:**
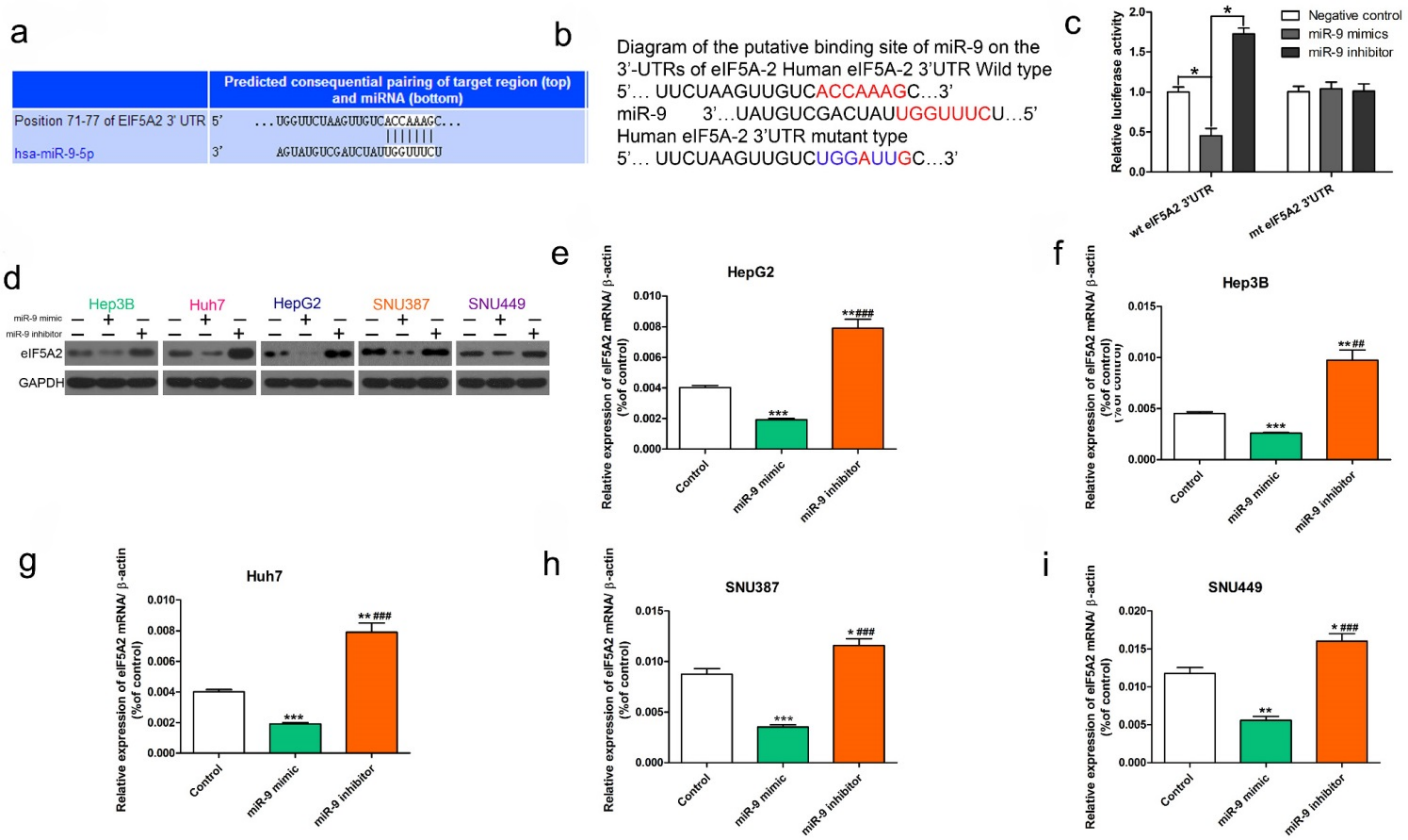
** miR-9 targeted *EIF5A2* in HCC cells.** (**A**) The predicted miR-9 binding site in the *EIF5A2* 3′ UTR. **(B)** miR-9 and its supposed binding sequence in the 3'-UTR of EIF5A2. For seeking region of miR-9, mutant binding site was acquired in the complementary site.** (C)** Plasmids which carried wild-type (wt) or mutant (mt) 3'-UTR of EIF5A2 and miR-9 mimic or miR-9 inhibitor were co-transfected to HEK293T cells. Luciferase activity was measured using a dual-luciferase reporter assay system (Promega) and normalized to Renilla activity. * vs. Negative control, P < 0.05. **(D)** HCC cell lines were transfected with miR-9 mimic, miR-9 inhibitor, or control for 48 h. The total protein was extracted and subjected to western blotting.** (E-I)** HCC cell lines were transfected with miR-9 mimic, miR-9 inhibitor, or control for 48 h. The total RNA was isolated and subjected to RT-qPCR. *vs. control, ^#^vs. miR-9 mimic, *P < 0.05, **P < 0.01, ***P < 0.001, *^##^*P < 0.01, *^###^*P < 0.001.

As previously reported, EIF5A2 may be a previous target of miR-9^26^, ^27^.We used the TargetScan Human database to determine the mechanism of miR-9-induced chemosensitivity and its possible target genes. EIF5A2, which has been implicated as an oncogene that enhances chemoresistance in several cancers, harbored a potential miR-9 binding site** (Figure 4A).** As shown in Figure 4 B, the “seed sequence” of miR-9 matched precisely with the 3'-UTR of EIF5A2. Next we showed evidence that supported the speculation. Obviously, the luciferase activity of wild-type (wt) EIF5A2 3'-UTR was decreased by increased miR-9 and was improved by reduced miR-9, while mutant (mt) EIF5A2 3'-UTR was not influenced (P < 0.05,** Figure 4C).**Transient transfection of miR-9 mimic in HCC cells significantly reduced EIF5A2 protein expression **(Figure 4D)**. Furthermore, qRT-PCR showed obviously decreased *EIF5A2* mRNA in miR-9 overexpression HCC cells compared to miR-9 knockdown HCC cells **(Figure 4E-4I)**. These results suggest that miR-9 regulates *EIF5A2* expression in HCC cells.

### EIF5A2 promoted cisplatin resistance in HCC cells

**Figure 5:**
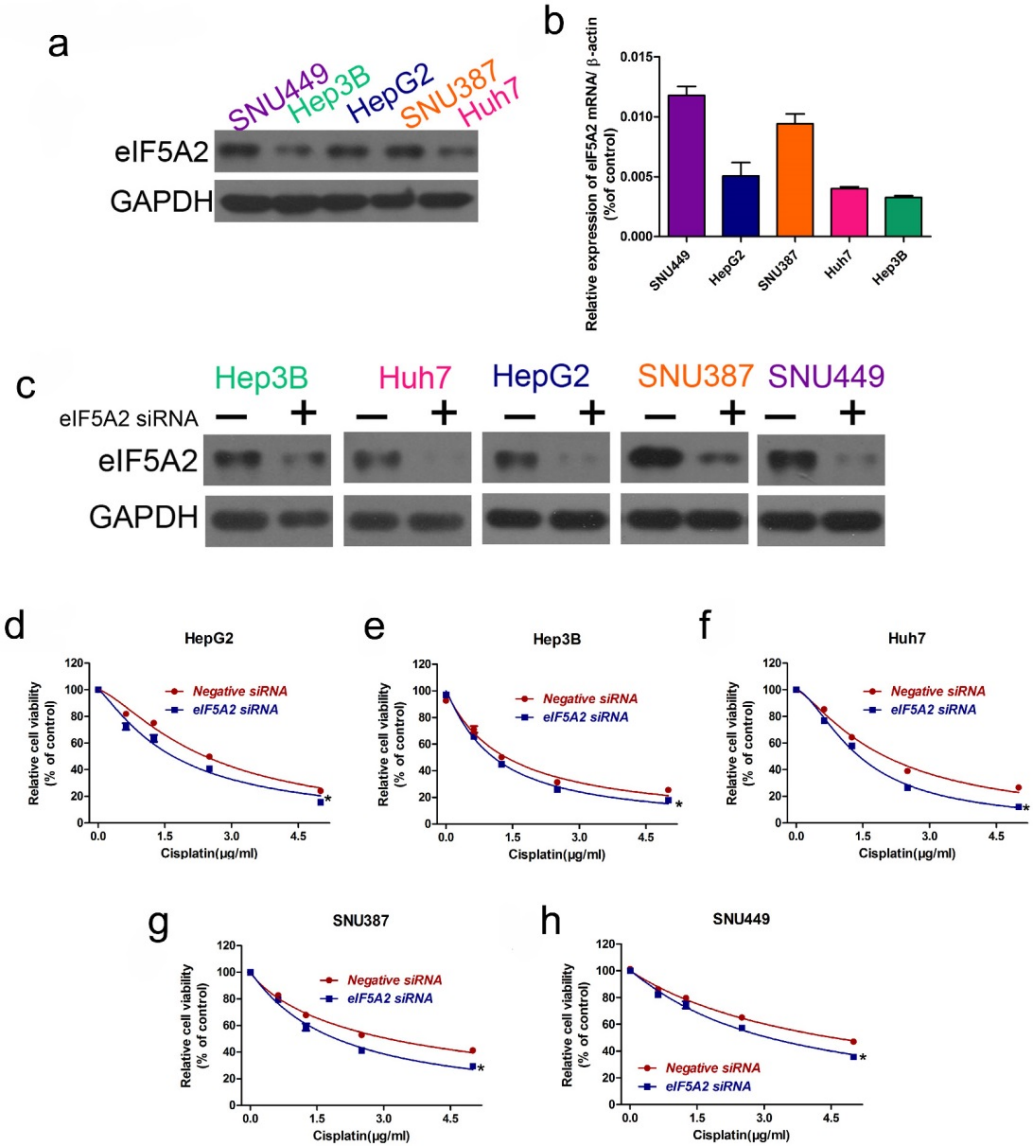
** EIF5A2 expression pattern in HCC cell lines and its effect on cisplatin sensitivity.** (**A, B**) Western blot (**A**) of EIF5A2 protein expression in HCC cell lines and its relative expression value as compared to GAPDH (**B**). (**C**) Western blot confirmation of EIF5A2 siRNA interference efficiency in HCC cell lines. (**D-H**) The effect of EIF5A2 on viability of HCC cells treated with EIF5A2 siRNA or negative siRNA in the presence of cisplatin (0, 1.5, 3.0, 4.5 μg/ml). *vs. Negative siRNA, *P < 0.05.

As *EIF5A2* is a target gene of miR-9, we investigated whether EIF5A2 contributes to cisplatin resistance in HCC cells. We examined EIF5A2 protein expression in the HCC cell lines, where the EIF5A2 protein expression trend was SNU449 > SNU387 > HepG2 > Huh7 > Hep3B, representing an opposite trend to that of miR-9 expression **(Figure 5A-B)**. Next, we used EIF5A2 siRNA to study EIF5A2 loss-of-function in HCC cells; western blotting confirmed the EIF5A2 siRNA interference efficiency** (Figure 5C)**. CCK-8 showed that EIF5A2 knockdown HCC cell lines had lower viability compared to the negative control siRNA group. These results suggest that EIF5A2 promotes cisplatin resistance in HCC cells.

### miR-9 promoted cisplatin sensitivity by downregulating EIF5A2 in HCC cells

**Figure 6:**
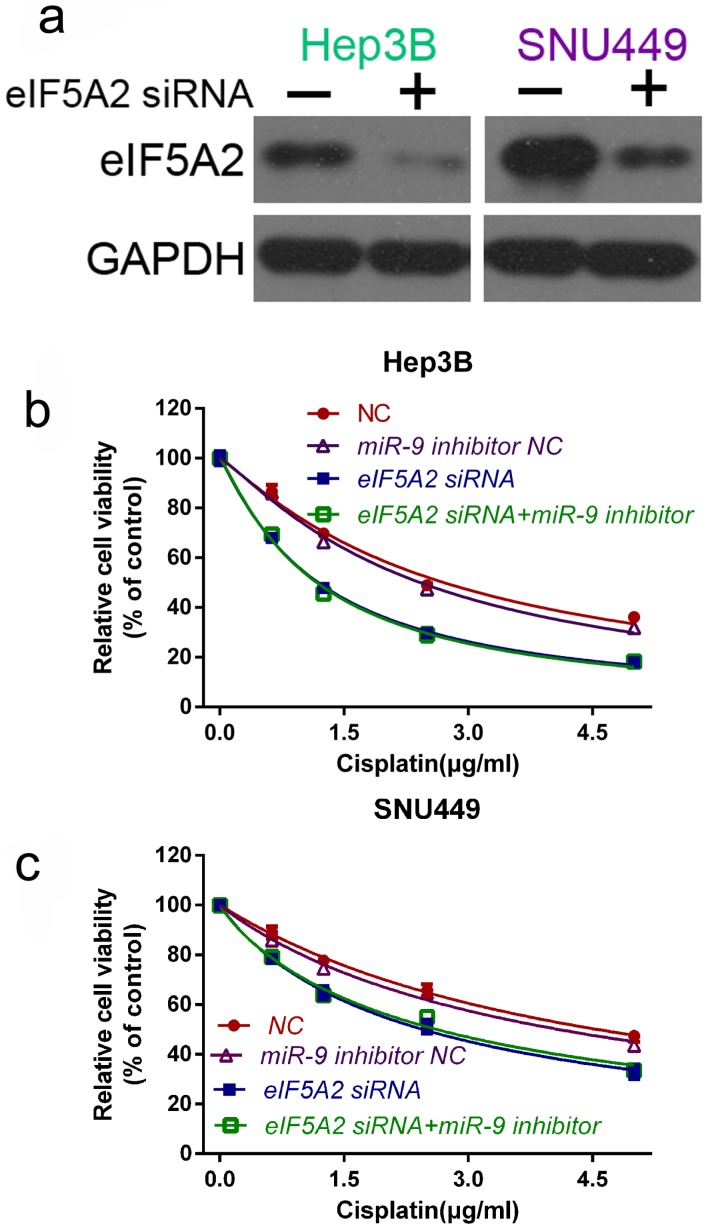
** EIF5A2 reversed the effects of miR-9 on cell viability.** (**A**) Western blot confirmation of EIF5A2 siRNA interference efficiency in Hep3B and SNU449 cells. (**B, C**) CCK-8 assay of Hep3B cells (**B**) and SNU449 cells (**C**) transfected with EIF5A2 siRNA plus synthetic miR-9 inhibitor or control in the presence of cisplatin (0, 1.5, 3.0, 4.5 μg/ml).

To further prove that EIF5A2 mediates miR-9-regulated cisplatin sensitivity , we compared the viability of Hep3B and SNU449 cells transfected with EIF5A2 siRNA or EIF5A2 siRNA plus miR-9 inhibitor in the presence of cisplatin. Western blotting confirmed the EIF5A2 siRNA interference efficiency** (Figure 6A)**. There was no difference in the cell viability between the EIF5A2 siRNA group and EIF5A2 siRNA plus miR-9 inhibitor group **(Figure 6B-C)**, indicating that EIF5A2 knockdown abolished the enhancement effect of the miR-9 inhibitor on cisplatin resistance. Taken together, these results indicate that EIF5A2 is involved in miR-9-mediated regulation of cisplatin sensitivity in HCC cells.

### The epithelial-mesenchymal transition (EMT) pathway was involved in miR-9- and EIF5A2-mediated regulation of cisplatin sensitivity in HCC cells

**Figure 7:**
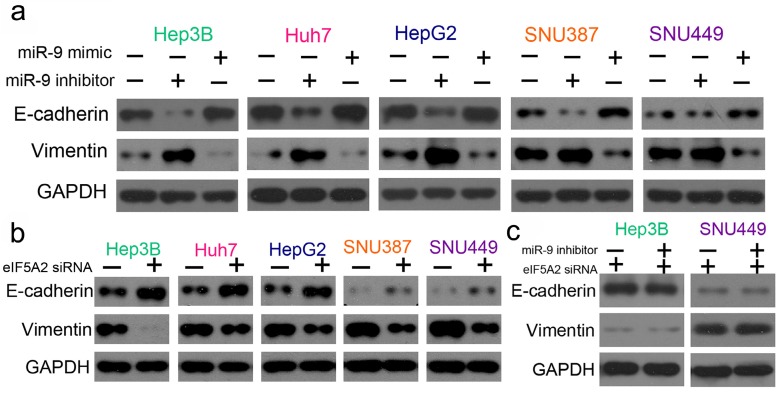
** miR-9 and EIF5A2 regulated the EMT pathway.** (**A**) Western blot comparison of E-cadherin and vimentin protein expression levels between HCC cells transfected with miR-9 mimic or miR-9 inhibitor. (**B**) Western blot comparison of E-cadherin and vimentin protein expression levels between HCC cells transfected with EIF5A2 siRNA or control siRNA. (**C**) E-cadherin and vimentin protein expression levels in Hep3B and SNU449 cells transfected with EIF5A2 siRNA alone or EIF5A2 siRNA plus miR-9 inhibitor. GAPDH was the internal control.

As EMT is one of the key events in tumor cell chemoresistance, we examined the epithelial markers (E-cadherin) and mesenchymal markers (vimentin) with western blotting. miR-9 overexpression signifycantly upregulated E-cadherin and downregulated vimentin, and the miR-9 inhibitor had the opposite effect** (Figure 7A).** As expected, EIF5A2 knockdown had the same effect on E-cadherin and vimentin expression as miR-9 overexpression **(Figure 7B).** When HCC cells were co-transfected with EIF5A2 siRNA and miR-9 inhibitor, the expression of E-cadherin and Vimentin showed no difference **(Figure 7C)**. Collectively, our results indicate that miR-9 may regulate cisplatin sensitivity by downregulating *EIF5A2* and inhibiting the EMT pathway.

## Discussion

HCC is the fifth most common malignancy worldwide, and long-term survival is poor. Chemoresistance is one of the major obstacles in the chemotherapeutic treatment of HCC 28, 29. Currently, the mechanisms underlying HCC chemoresistance are largely unknown. Therefore, it is urgent to identify novel mechanisms in patients with HCC who are resistant to chemotherapy. miRNAs are involved in chemoresistance in many cancers 30. In the present study, we focused on miR-9, which has been indicated as an important miRNA in cancer development. It has been reported that miR-9 could enhance sensitivity to cisplatin of NSLSC through inhibiting EIF5A2 expression^26^. Furthermore, miR-9 could enhance the chemosensitivity to daunorbicin in AML cells via regulating EIF5A2^27^. As an oncogene or a tumor suppressor, MiR-9 also plays an important role in migration and invasion in HCC cells. Accumulated evidence has indicated that over-expression of miR-9-5p could enhance cell migration and invasion while miR-9-3p could inhibit cell migration an invasion in HCC cells^20^, ^31^. However, in this study, we revealed that miR-9-5p was a suppressor, we had examined the level of miR-9 and found that it was lower in cancer tissue than that in normal tissue and up-regulation of miR-9 could enhance sensitivity to cisplatin in HCC cells. Xue et al had reported that over-expression of miR-9-5p could enhance sensitivity to cetuximab in epithelial phenotype HCC cells^32^, which is consistent with our results.

Here, HCC cells with high miR-9 expression had low cell viability under cisplatin treatment. The cell viability of the five HCC cell lines followed a trend opposite to their miR-9 levels. To further explore the effect of miR-9 on cisplatin sensitivity in HCC, we employed gain- and loss-of-function approaches *in vitro* and *in vivo*. miR-9 overexpression in HCC cells reduced proliferation and promoted apoptosis *in vitro* and *in vivo*, which supports the idea that miR-9 is a potent cisplatin sensitizer in HCC. This result is consistent with that of Sun et al. on ovarian cancer, where miR-9 enhanced sensitivity to cisplatin and poly(ADP-ribose) polymerase (PARP) inhibition 33.

We used TargetScan Human to determine the potential mechanism of miR-9-induced chemosensitivity and its possible target genes, and found that *EIF5A2*, an oncogene, was in the list. *EIF5A2*, an essential component of translation elongation, is a novel oncogene and a potential molecular target in human cancer 34, 35. *EIF5A2* upregulation has been reported in many human cancers, such as cervical cancer 36, HCC 37, bladder cancer 38, and gastric cancer 39. It was recently reported that inhibiting *EIF5A2* enhances the chemosensitivity of pancreatic ductal adenocarcinoma cells to gemcitabine 40, non-small cell lung cancer (NSCLC) cells to cetuximab 41, and esophageal squamous cell carcinoma cells to 5-FU 42. In HCC, EIF5A2 is a target of GC7 (N1-guanyl-1,7-diaminoheptane), and GC7 downregulation of EIF5A2 sensitizes HCC cells to doxorubicin 43, 44. In the present study, we prove that *EIF5A2* is a target gene of miR-9 in HCC, and further prove that *EIF5A2* knockdown sensitizes HCC cells to cisplatin. EIF5A2 siRNA transfection confirmed that *EIF5A2* knockdown reverses the cell viability increased by miR-9 inhibitor. These results indicate that miR-9 enhances chemosensitivity of HCC cells, at least partially, by downregulating *EIF5A2*.

Increasing evidence demonstrates that EMT plays crucial roles in acquired drug resistance in many cancers, including HCC 45. EIF5A2 and miR-9 have been associated with EMT progression in HCC 20, 46, 47. However, the underlying relationship among chemosensitivity, EIF5A2, miR-9, and EMT is unknown. Here, we prove that miR-9 overexpression inhibits EMT in HCC by downregulating *EIF5A2* expression. The EMT pathway may be involved in miR-9-mediated regulation of cisplatin sensitivity.

In conclusion, we demonstrate that miR-9 overexpression sensitizes HCC cells to cisplatin by inhibiting *EIF5A2* and preventing EMT, which sheds light on the mechanisms of miR-9 regulation of HCC chemosensitivity and provides a novel target for improving chemotherapy efficiency in HCC.

## Supplementary Material

Supplementary figures and tables.

## Figures and Tables

**Table 1:** IC50 values and statistical analyses of cisplatin treatments in HCC cell lines.

HCC cell line	IC_50_ (μg/mL)^▲^
	Cisplatin(Cis)
HepG2	2.491±0.1002
Hep3B	1.356±0.07875
Huh7	1.706±0.04817
SNU387	3.104±0.1047
SNU449	4.556±0.2424

IC_50_ values show concentrations of Cisplatin [μg/mL; mean (95% confidence intervals)]

**Table 2:** The viability of HCC cells transfected with miR-9 mimic or miR-9 inhibitor combined with different concentrations of cisplatin.

cell lines	IC_50_(μg/mL)			
	Control	NC	miR-9 mimic+Cis	miR-9 inhibitor+Cis
HepG2	2.340±0.08808	2.186±0.1220	1.526±0.03643*	3.175±0.1491*
Hep3B	1.576±0.07519	1.610±0.08595	1.231±0.05804*	2.306±0.07507*
Huh7	1.825±0.08757	2.030±0.05761	1.237±0.03390*	3.044±0.07954*
SNU387	3.104±0.1047	3.286±0.1060	2.089±0.1014*	6.2816±0.6380*
SNU449	4.553±0.2724	4.528±0.2903	2.540±0.1046*	6.778±0.4267*

IC_50_ values show Cisplatin concentration [μM. mean (95% confidence intervals)]. *P<0.05 vs Control
