# Supplementary material for: Overexpression of microRNA-9 enhances cisplatin sensitivity in hepatocellular carcinoma by regulating EIF5A2-mediated epithelial-mesenchymal transition
Source: Int J Biol Sci. 2020 Jan 16;16(5):827–37. doi: 10.7150/ijbs.32460 (PMC7019138; doi:10.7150/ijbs.32460)
Supplement: Supplementary file 1 — Supplementary figures and tables. [file ijbsv16p0827s1.pdf]

Overexpression of microRNA-9 enhances cisplatin sensitivity in hepatocellular carcinoma by regulating EIF5A2-mediated epithelial–mesenchymal transition

Ying BAO[1*],Yibo ZHANG[2*],Yongliang LU[3*], Huihui GUO[1*] , Zhaohuo DONG[1], Qiuqiang CHEN[1], Xilin ZHANG[1], Weiyun SHEN[1],Wei CHEN[4], Xiang WANG[1]

[1]Key Laboratory for Translational Medicine, First Affiliated Hospital, Huzhou University, the First People's Hospital of Huzhou, huzhou 313000,China

[2]Department of Hepatobiliary and Pancreatic Surgery, Second Affiliated Hospital of Zhejiang University School of Medicine, Hangzhou, 310009, China

[3]Department of medicine,Huzhou University, huzhou 313000,China

[4]Institute of Molecular Engineering, University of Chicago, Chicago, 60637, USA.

* Contributed equally to this work

Address corresponding to:

Xiang WANG, First Affiliated Hospital,Huzhou University, the First People's Hospital of Huzhou, huzhou 313000, China .

Tel: +86572-2023728; Fax: +86572-2023728.

E-mail address:wangx2017@126.com

Wei Chen, Institute of Molecular Engineering, University of Chicago, Chicago, 60637, USA

Tel: +1 8722128084; Fax+1 7737027752

E-mail address: weichen@uchicago.edu

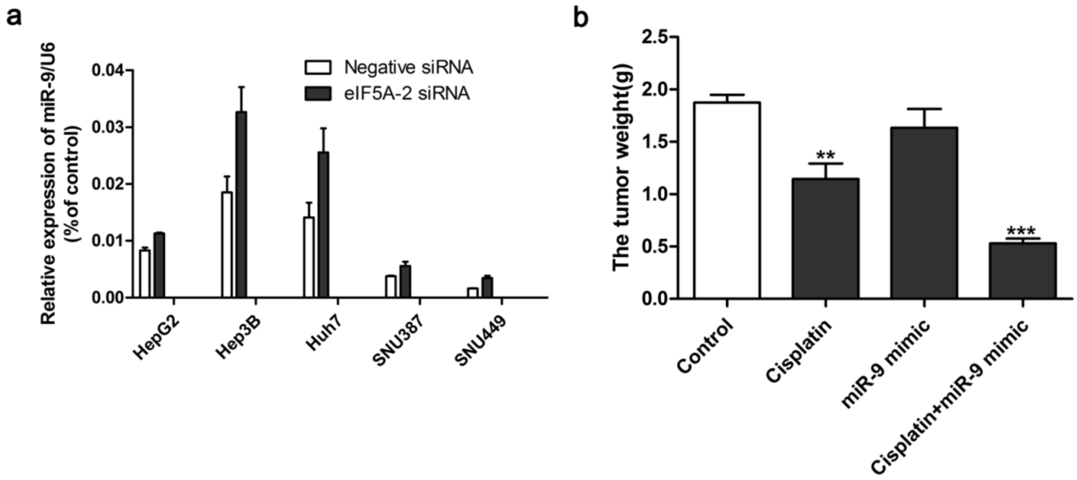

Figure S1

a. Real-time PCR was determined to detect the level of miR-9 after EIF5A2 knockdown or not. **b**. Tumor weight of xenograft tumors treated with control, cisplatin, miR-9 mimic, or cisplatin plus miR-9 mimic (n = 4 per group). **P < 0.01,***P < 0.001 vs Control.

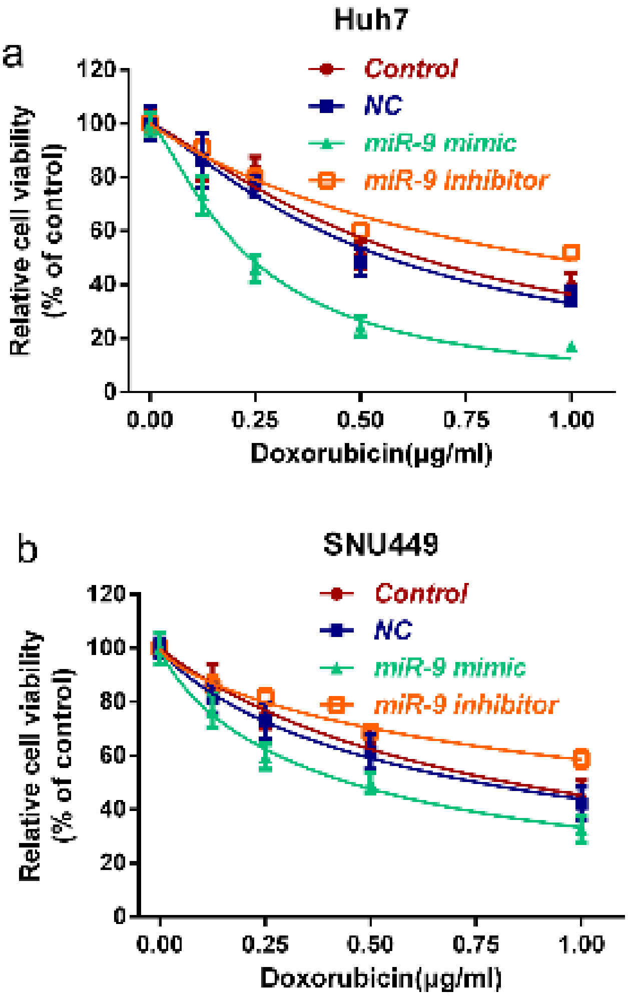

Figure S2

a-b. CCK-8 determined the sensitivity to doxorubicin after transfected with miR-9 mimic, inhibitor, control or NC in HCC cells.

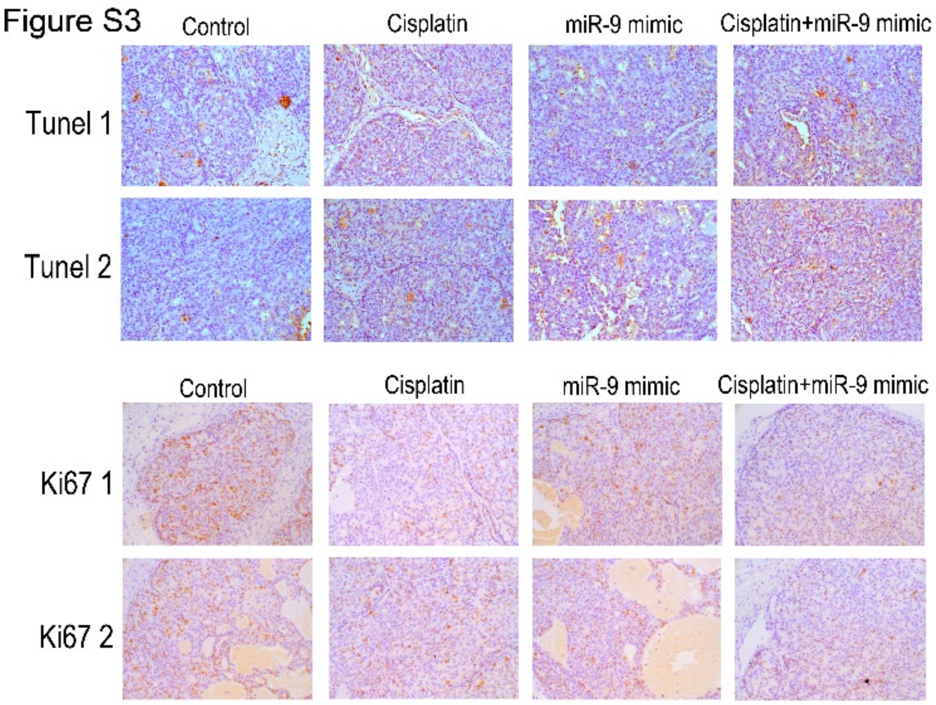

Figure S3

TUNEL of apoptosis in the treatment groups and Ki-67 staining in the treatment groups and rate of Ki-67 positive staining.
